# Simple Modification of Karl-Fischer Titration Method for Determination of Water Content in Colored Samples

**DOI:** 10.1155/2012/379724

**Published:** 2012-03-19

**Authors:** Eva Tavčar, Erika Turk, Samo Kreft

**Affiliations:** Faculty of Pharmacy, University of Ljubljana, Aškerčeva cesta 7, 1000 Ljubljana, Slovenia

## Abstract

The most commonly used technique for water content determination is Karl-Fischer titration with electrometric detection, requiring specialized equipment. When appropriate equipment is not available, the method can be performed through visual detection of a titration endpoint, which does not enable an analysis of colored samples. Here, we developed a method with spectrophotometric detection of a titration endpoint, appropriate for moisture determination of colored samples. The reaction takes place in a sealed 4 ml cuvette. Detection is performed at 520 nm. Titration endpoint is determined from the graph of absorbance plotted against titration volume. The method has appropriate reproducibility (RSD = 4.3%), accuracy, and linearity (*R* ^2^ = 0.997).

## 1. Introduction

Researchers dealing with development of pharmaceutical and food products are often confronted with the need for water determination in their samples. For research or manufacturing purposes, several methods for water determination have been developed [1, 2]. The selection of the most appropriate method depends on whether the sample is in solid or liquid form, time, and available equipment. One of the most frequently used laboratory-scale methods for determination of water content in liquid samples is bipotentiometric Karl-Fischer (KF) titration. It is based on the oxidation of sulphur dioxide by iodine with consumption of water: I_2_ + 2H_2_O + SO_2  _ → 2HI + H_2_SO_4_. The endpoint of a titration represents a volume of the titrant needed for the reaction with the total amount of water present in the sample. Variations of the basic titration principle are being constantly developed. Some samples are not suitable for KF analysis due to their insolubility and chemical interaction of the sample with KF reagents. Solvent is chosen with respect to stability, reaction rate, conductivity, side reactions, and solvation of the sample in the reagent solution. Ketones and aldehydes interfere with basic KF titration because they react with methanol to form water, causing incorrectly high water results and vanishing endpoints. The problem can be resolved by utilizing methanol-free reagents that suppress side reactions. Ethanol, octanol, ethylene glycol, formamide derivatives, pyridine, acetonitrile, tetrahydrofuran, and ethylene/ethyl methyl carbonate or their mixtures were also utilized [3–12]. In KF titration, the endpoint of a titration can be determined by visual observation of color or by electrometry (coulometry or volumetry) [13–17].

The improvement of KF titration techniques led to the development of KF titrators, precise but costly apparatus, usually available in specialized laboratories only. For standard coulometric titrations, smaller compartment with catholyte solution is immersed in the main anode compartment of the titration cell, which contains the sample dissolved in anolyte (iodide and a sulphur dioxide/base buffer). Iodine is generated electrochemically and consumed for water titration. Constant current is detected bipotentiometrically by two detector electrodes. At the equivalence point, excess iodine abrupts voltage drop. The amount of water in the original sample is calculated through the amount of current needed to generate iodine and reach the endpoint. The volumetric titration is based on the same principles except that the anode solution is used as the titrant solution [9, 11, 18–27].

Apart from KF titration, other possible methods can be divided by the principle of water determination. Water can be physically separated from the sample by distillation, chromatography, or oven infrared or microwave drying. Those methods are being widely used as they require casual equipment, but are time consuming and often unspecific due to other volatile substances present in the sample [31–36]. As water content influences physical properties of the sample, it can also be determined through densitometric, polarimetric, refractometric, or electrical methods [28–30]. Densitometric method utilizing Eichhorn-type hydrometer is an example of a simple method, but specific only for honey samples [28, 29, 31–33]. NMR, microwave, and NIR spectroscopy and microwave resonator methods base on measurement of characteristic properties of the water molecule. In comparison to KF titration, they do not produce waste and are capable of simultaneous prediction of other sample properties. Moreover, as nondestructive methods, they enable in-process measurements. On the other hand, they sometimes lack the ability of bound water determination and require extremely product-specific calibration [21, 32, 34–43]. All those methods are appropriate for samples that introduce large errors in determination by KF titration, for example, because of additives in lubricating oils or insolubility of samples in KF reagents [38, 44, 45].

Several studies, comparing different water determination methods, were carried out, often recognizing KF titration as the simplest, the most accurate, and reproducible among them and therefore used as a reference method in many studies [46–56]. Recently, KF two-component titration technique for water determination in lactose obtained international standard, replacing less selective gravimetric oven drying method that based on mass loss of all volatiles in powder samples [57].

Simple volumetric kits, suitable for use without KF titrator, are increasingly available on the market. In one packing, the kits usually include all the equipments and reagents needed. The endpoint of a titration is determined by visual change of color from colorless to yellow and is suitable for water determination in colorless samples [58]. During preparation of liquid plant extracts or syrups, water is one of the components with an important effect on their physical and chemical properties. Plant extracts and syrups are usually complex mixtures of different colored components, masking the change of color to yellow at the titration endpoint. The aim of this research was to determine the water content in brown polyethylene glycol extract in laboratory scale, with no specialized equipment available. Simple volumetric kit was tried out, but the endpoint of a titration was impossible to visually observe due to the original color of the sample. The application of UV/VIS spectrophotometric detection proved to be the right solution of the problem.

## 2. Materials and Methods

### 2.1. Preparation of Samples

*Abies alba* bark extract (AABE) was prepared in polyethylene glycol 400 (PEG) solution. It was composed mostly of polyphenols and tannins. AABE was a dark brown liquid with a density of 1,145 kg/dm^3^. PEG (Panreac) was used as a reference sample. Deionized water was added to pure AABE and pure PEG to obtain samples with 4 and 7.5% added water.

### 2.2. Karl-Fischer Titration with Visual Observation of Color Change at Endpoint

Commercially available HYDRANAL-Moisture Test Kit from Sigma-Aldrich was used for reference moisture determination. All the equipments needed were provided with the kit. It consisted of two-component ethanol-based reagents: HYDRANAL Solvent E, working medium containing imidazole, sulphur dioxide and diethanolamine, and HYDRANAL Titrant E containing iodine. According to manufacturer's instructions for use, 20 mL of HYDRANAL Solvent E was added to the titration vessel, which was then tightly sealed with a septum. The titrant was delivered through the septum with a syringe to react with the present water, until a color change from colorless to yellow occurred. A known amount of sample was added by a syringe to the titration vessel via septum, and a newly filled titration syringe was used for a new titration until the color change from colorless to yellow occurred. Consumption volume of the titrant was read off the titration syringe. The water content, in percent by volume, was calculated from the consumption of the titrant and the sample volume.

### 2.3. Karl-Fischer Titration with Spectrophotometric Measurement of the Endpoint

**Figure 1:**
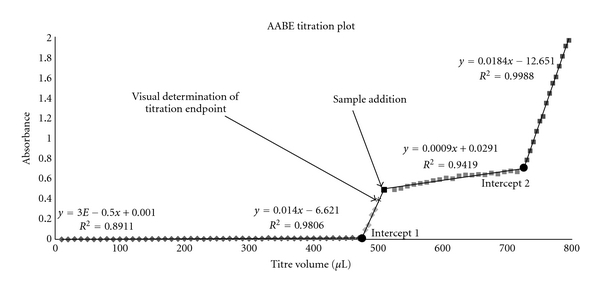
AABE titration plot, recorded at 520 nm.

The reagents from previously described commercial kit were used for optimized procedure: 2 mL of the HYDRANAL Solvent E and a small magnet were added to a 4 mL glass cuvette 1000 QS. The inner atmosphere of the cuvette was isolated from the surroundings using latex foil (cut finger top of a Vileda 100% latex extra soft protective glove) fixed to a cuvette with duct tape. Spectrophotometer PerkinElmer LambdaBio+ was used for obtaining absorbance of sample solutions at 520 nm wavelengths. The absorbance of pure solvent was recorded as a background. Exact volumes of HYDRANAL Titre Component were being added slowly into the cuvette using a fixed syringe (100 *μ*L < ±1% borosilicate glass), and the mixture was stirred with magnetic stirrer. Absorbance was measured after each addition of 10 *μ*L of the titrant, until the three values deviated from zero (intercept 1). A new 100 *μ*L syringe was used for addition of the exact volume of a sample into the cuvette. Absorbance was measured after each addition of 5 or 10 *μ*L of the titrant. Microsoft Office Excel was used for data processing. Absorbance values were plotted against volumes of the added titrant, as shown in Figure 1. Four lines were fitted into the data using linear regression. The intercept between the first and second lines (intercept 1) shows the titrant volume required to react with water, present in the system (solvent), before the sample addition. The intercept between the third and fourth lines (intercept 2) shows the titrant volume required to react with water in the system after the sample addition. The difference between the volume at intercept 1 and intercept 2 represents the accurate titrant volume at the endpoint of titration. In conventional method, consumption of the excess of titrant at the moment right after sample addition would decrease the absorbance value to zero. Due to the colour of our sample, the absorbance of working solution at that time is not zero. By coincidence, the absorbance is very similar to the one of overtitrated working solution. In other words, decrease of absorbance because of the moisture, added with the sample, is masked by the colored substances, present in the sample.

## 3. Results and Discussion

Modified method for moisture determination is based on HYDRANAL-Moisture Test Kit, with several modifications in the applied volumes and the detection of the titration endpoint. The main advantage of the method is suitability for fast and simple water determination of colored samples, without the need for complex and expensive laboratory equipment. Accurate absorbance measurement has been employed, using spectrophotometer instead of visible observation. The system was therefore adjusted to a smaller cuvette, replacing bigger glass bottle. The procedure consequently required lower consumption of reagents and samples.

### 3.1. Correction of the Solvent Titration

During each analysis, certain amounts of titrant needed to be added for solvent neutralization before the sample addition. After reaching this first endpoint, the absorbance rose to a value corresponding to the surplus of the titrant volume. In conventional method, the titration would stop when the first color change was observed. The difference between the actual endpoint (intercept 1) and the point, at which the color is observed, represents an error in accuracy and repeatability. This difference was cca. 15 *μ*L, which can, depending on the total titrant consumption, represent more than 10% error of the final result. This problem was solved by plotting linear regression line of obtained absorbance values against added titrant volumes, and extrapolating the line to zero (intercept 1) for determination of the actual titration endpoint. When the intention of the moisture measurement is only to determine the approximate value and more than 10% error is acceptable, spectrophotometric correction at solvent titration can be skipped. In this case, the first endpoint can be determined visually, since the solvent is not colored.

### 3.2. Method Validation

Repeatability of the modified KF titration method with spectrophotometric measurement of the endpoint for analyses of colored samples was tested by analyzing the water content in an AABE sample five times and PEG samples three times. Measurements of AABE were carried out in a three-day period for intermediate repeatability evaluation. Results are shown in Table 1.

**Figure 2:**
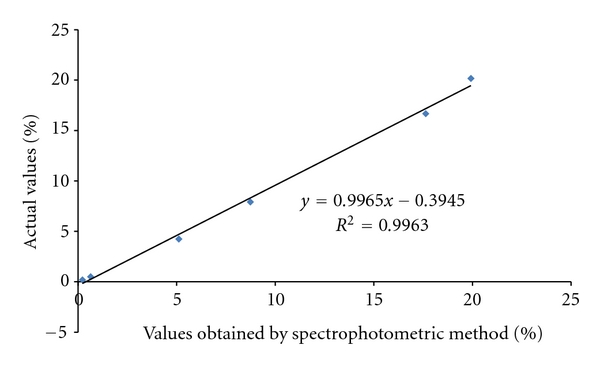
Spectrophotometric method values plotted against actual values of moisture.

As shown in Table 2, a set of six samples was analyzed to assess method accuracy and linearity. The obtained values were compared to actual values, which were estimated in different ways. Sample nos. 1 was HYDRANAL Standard 5.00 with 0.5% moisture, provided by the manufacturer of the kit. Its water content was declared on the label (0.500%) and determined by the reference method (0.522%). Samples nos. 2 and 3 were prepared by adding known amounts of water (4 and 7.5%) to AABE samples and samples no. 4 and 5 to pure PEG. Water content in sample 6 (pure PEG) was determined by the reference method (HYDRANAL kit). All measurements corresponded very well to the predicted values. The linearity of the spectrophotometric method can be observed from Figure 2.

Theoretical detection limit of the method is shown in Table 3. Recommended amounts of sample are estimated from the minimum titrant consumption, which is limited by relatively higher error for small volumes, and maximum titrant consumption, which is limited with the cuvette volume.

For comparison, the original HYDRANAL-Moisture Test Kit method was tried out. Standard was analyzed for five times, giving an average value of 0.522% (Table 4). As pure PEG solvent is a colorless sample, it was also analyzed using original method. Seven analyses gave an average moisture value of 0.188%. In AABE sample, its yellow color masked the titration endpoint; therefore, a measurement with the original method could not be performed.

### 3.3. Method Development

Many variables were changed during method development. The water content of a sample could be influenced by the relative humidity of the laboratory, which could lead to biased property values. Good isolation of the system is therefore critical for proper moisture determination. Several ideas were tried out to make the solvent and the sample isolated from surroundings, such as sealing the cuvette with parafilm, duct tape, rubber plug and silicon, and foam ear plugs. The most effective was a cut finger of a glove fixed with duct tape. Optimum glove type was Vileda 100% latex extra soft protective gloves that were thicker than several tested laboratory gloves, which were easily broken, when being punctured with a syringe needle. The wavelength choice of the spectrophotometric detection was tested at 420, 440, 460, 480, 500, and 520 nm. Measuring at 520 nm enabled absorbance measurement after addition of 50 *μ*L of titrant surplus, while at lower wavelengths, the absorbance was too high to be measured. This wavelength corresponded to the choice of wavelength reported in literature [11]. Time needed for a single analysis was shortened by skipping the correction of the solvent titration, which led to a higher standard deviation. A spectrophotometric method without the correction of the solvent titration would therefore be applicable when quick, but less accurate analyses are needed.

## 4. Conclusion

We developed a method enabling moisture determination in colored samples, using simple equipment. The method has appropriate reproducibility, accuracy, and linearity. The main drawback of the method is the measurement of small volumes, so results of every analysis can be affected by human or laboratory equipment error. Hence, the precision of the analyst and careful choice of the equipment are indispensable. Those facts give the analyzer the ability to implement the analysis according to his needs, balancing between the accuracy of the results and time consumption. 

## Figures and Tables

**Table 1:** AABE moisture determination series.

Analysis no.	Day	Measured water content (%)		
		AABE	PEG + 4%	PEG + 7.5%
1	1	13.322	5.170	8.655
2	1	12.152	5.204	8.350
3	1	12.079	4.940	9.192
4	2	13.304	/	/
5	3	12.504	/	/
Average		12.672	5.105	8.732
Relative standard deviation		4.282	2.813	4.882
Standard deviation		0.543	0.144	0.426

**Table 2:** Spectrophotometric method values and actual values of moisture in set of samples.

Sample no.	Samples	Values obtained by spectrophotometric titration method (%)	Actual values	Absolute error
1	HYDRANAL standard 5.00	0.634	0.500 (0.522)	0.134 (0.112)
2	AABE + 7.5% water	19.919	20.172	0.253
3	AABE + 4% water	17.627	16.672	0.955
4	PEG + 7.5% water	8.732	7.920	0.813
5	PEG + 4% water	5.105	4.238	0.867
6	PEG	0.220	0.188	0.032

**Table 3:** Theoretical detection limit with sample volume recommendations.

Water content (%)	Sample volume (*μ*L)	Titre consumption (*μ*L)
5–25	5	50–250
2.5–12.5	10	50–250
1.25–6.25	20	50–250
0.62–3.13	40	50–250
0.31–1.56	80	50–250
0.21–1.04	120	50–250
0.16–0.78	160	50–250
0.10–1.04	120	25–250

**Table 4:** Accuracy of moisture determination with the original method with visual observation of the endpoint.

Sample	Number of analyses	Average moisture value	SD	RSD
PEG	7	0.188	0.016	8.278%
Standard	5	0.522	0.016	3.065%
